# A fly’s eye view of quiescent neural stem cells

**DOI:** 10.1093/oons/kvac001

**Published:** 2022-05-04

**Authors:** Mahekta R Gujar, Hongyan Wang

**Affiliations:** Neuroscience & Behavioral Disorders Programme, Duke-NUS Medical School, 8 College Road, 169857, Singapore; Neuroscience & Behavioral Disorders Programme, Duke-NUS Medical School, 8 College Road, 169857, Singapore; Department of Physiology, Yong Loo Lin School of Medicine, National University of Singapore, 117597, Singapore; NUS Graduate School for Integrative Sciences and Engineering, National University of Singapore, 28 Medical Drive, 117456, Singapore

**Keywords:** neuropil, cellular protrusion, reactivation, quiescence, neural stem cells, Drosophila

## Abstract

The balance between proliferation and quiescence of stem cells is crucial in maintaining tissue homeostasis. Neural stem cells (NSCs) in the brain have the ability to be reactivated from a reversible quiescent state to generate new neurons. However, how NSCs transit between quiescence and reactivation remains largely elusive. *Drosophila* larval brain NSCs, also known as neuroblasts, have emerged as an excellent *in vivo* model to study molecular mechanisms underlying NSC quiescence and reactivation. Here, we discuss our current understanding of the molecular mechanisms underlying the reactivation of quiescent NSCs in *Drosophila*. We review the most recent advances on epigenetic regulations and microtubule cytoskeleton in *Drosophila* quiescent NSCs and their cross-talk with signaling pathways that are required in regulating NSC reactivation.

Neural stem cells (NSCs) are crucial for the development, regeneration and repair of the nervous system. Most NSCs in the mammalian adult brain exist in a quiescent or mitotically dormant state [1–3]. Quiescent NSCs can re-enter the cell cycle (reactivate) to generate new neurons in response to various physiological stimuli, such as injury, the presence of nutrients and physical exercise [3–10]. Conversely, stress, anxiety and old age greatly reduce the proliferation capacity of NSCs [11]. Dysregulation of NSC quiescence and reactivation severely affect tissue homeostasis [12, 13]. NSCs in invertebrates such as *Drosophila melanogaster* also switch between a reversible transition between quiescence and reactivation [14–17]. *Drosophila* NSCs, also known as neuroblasts, in the central brain and thoracic ventral nerve cord (VNC) enter into quiescence at the end of embryogenesis and exit quiescence (termed reactivation) largely within 24 hours upon larval hatching in response to feeding [14–19]. Dietary amino acids stimulate the TOR-kinase pathway in the fat body, which presumably induces the synthesis of unknown fat-body–derived signals (FDSs) that are thought to reach the brain and VNC, stimulating NSC reactivation [20–23].

The *Drosophila* brain is separated from the blood-like hemolymph by a functional analogue of blood–brain barrier (BBB) that acts as an insulation barrier to protect the CNS [24]. Recent work from the Brand lab showed that in early larval stages, NSCs are not covered by the cortex glial membrane, allowing direct contact between the BBB glia and NSCs [25]. Thus, BBB glia provides an important niche for the regulation of NSC quiescence and reactivation via various signaling pathways. In response to nutrition, insulin/insulin-like growth factor (IGF) signaling (IIS) controls growth, metabolism and longevity [26]. The function of IIS in regulating growth is evolutionarily conserved in *Drosophila* and mammals. *Drosophila* contains a single insulin/IGF receptor (dInR) and eight insulin/IGF-like peptides (dILPs 1–8) [27, 28]. The dILPs secreted from the BBB glia act locally by directly activating the InR/phosphatidylinositol 3-kinase (PI3K)/Akt pathway as well as the TOR pathway in underlying NSCs [23, 29].

Apart from promoting NSC reactivation, the *Drosophila* BBB glia niche also expresses and secretes factors that maintain NSC quiescence. In the absence of dietary amino acids, BBB glia express intercellular transmembrane proteins Crumbs and Echinoid that activate the Hippo pathway, keeping NSCs in quiescence [30–34]. Recent studies have identified several regulators of signalling pathways that are critical for NSC quiescence and reactivation. One such intrinsic regulator of the InR pathway is the Heat shock protein 83 (Hsp83), an Hsp90 family molecular chaperone that promotes NSC reactivation intrinsically by an association with InR [35]. The Cullin-RING ligase, CRL4^Mahjong^, an evolutionarily conserved E3 ubiquitin ligase composed of Cullin4, DDB1, Roc1 and a substrate receptor named Mahjong, downregulates the Hippo pathway [36]. STRIPAK complex members function as an intrinsic molecular switch coordinating Hippo and InR/PI3K/Akt pathways, first maintaining NSC quiescence and subsequently triggering NSC reactivation [37].

How is NSC reactivation coupled with nutrient requirements? During the embryonic to larval transition, although most NSCs are in quiescence, four mushroom Body (MB) NSCs and one lateral NSC in the central brain continue dividing, independent of nutrients [20, 38]. In contrast to the PI3K-dependent reactivation in non-MB NSCs, PI3K is dispensable for MB NSC proliferation [38]. This nutrient- and PI3K-independent MB NSC proliferation requires the transcription factor Eyeless (Ey), a Pax-6 orthologue expressed primarily in MB NSCs [38]. Ey appears to bind to regulatory regions of genes involved in metabolism [38]. In the future, it will be important to investigate the metabolism signatures of MB vs non-MB NSCs.

Two recent comprehensive reviews have covered conceptual progress on quiescent NSCs from different model systems [9, 10]. In this review, we will focus on the most recent advances in epigenetic regulations and microtubule cytoskeleton in *Drosophila* quiescent NSCs that were not previously discussed (Figure 1).

**Figure 1:**
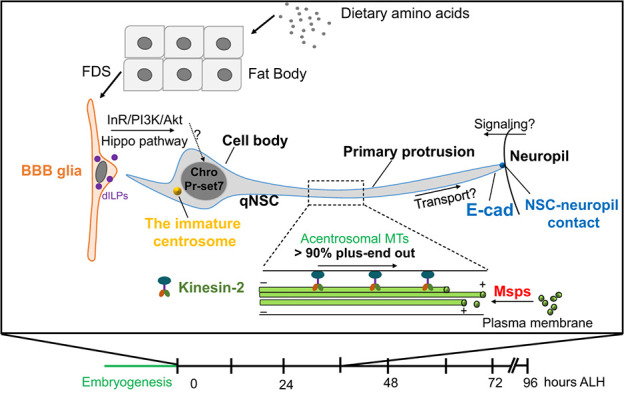
Diagrammatic representation showing various factors regulating *Drosophila* NSC reactivation. Dietary amino acids are sensed by the fat body that generates mitogens, stimulating blood–brain barrier glial cells to secrete insulin-like peptides (dILPs). dILPs activate the InR/PI3K/Akt pathway in underlying NSCs and promote their reactivation, while the Hippo pathway maintains NSC quiescence. The spindle matrix complex containing Chromator (Chro) functions downstream of the InR/PI3K/Akt signaling pathway as a key intrinsic regulator of NSC reactivation. The H4K20 monomethyl transferase, Pr-set7, is also required for the reactivation of *Drosophila* NSCs. In the primary protrusion of quiescent NSCs, microtubules are predominantly acentrosomal and oriented plus-end-out. Mini spindles (Msps) and Kinesin-2 promote NSC cell cycle re-entry and target E-cadherin to NSC-neuropil contact during NSC reactivation. ALH, after larval hatching; BBB, blood–brain barrier; Chro, Chromator; dILPs, insulin/IGF-like peptides; E-cad, E-cadherin; FDS, fat-body–derived signal; InR, Insulin receptor; Msps, mini spindles; MTs, microtubules; PI3K, Phosphatidylinositol 3-kinase; qNSC, quiescent NSC.

## TRANSCRIPTIONAL AND EPIGENETIC REGULATIONS OF NSC REACTIVATION

Several transcription factors and spatial regulators such as the Hox protein play a role in NSC reactivation and quiescence [18]. The homeodomain transcription factor Prospero (Pros) is also capable of driving proliferating NSCs into quiescence when transiently expressed in NSCs [39]. Pros is repressed by spindle matrix proteins composed of Chromator (Chro)/Chriz, Megator and enhanced adult sensory threshold (East) that function intrinsically in NSCs [40]. Chro also activates *grainy head* (*grh*), a temporal transcription factor in NSCs, and probably functions downstream of the InR/PI3K/Akt pathway to promote NSC reactivation [40]. Future studies will determine whether Chro has a direct link with the InR/PI3K/Akt pathway during NSC reactivation.

Epigenetic regulators that control histone modifications or chromatin remodeling also play important roles in regulating NSC behaviors [41]. Recent work demonstrated the importance of *Drosophila* KMT5A/Pr-set7/SETD8, the sole histone H4 Lys 20 monomethyltransferase (H4K20me1), in NSC reactivation [42]. KMT5A/Pr-set7/SETD8 plays critical roles in maintaining genome stability, DNA repair and replication, cell cycle regulation, and chromatin compaction [43, 44]. Increasing evidence suggests that variants of histone lysine methyltransferases including KMT5A are associated with neurodevelopmental disorders [45]. In *Drosophila*, loss of *pr-set7* resulted in delayed NSC reactivation [42]. Though the role of H4K20me1 as an activator or repressor has been debatable, targeted DNA adenine methyltransferase (Dam) identification (TaDa)-based *in vivo* profiling, demonstrated that Pr-set7 can bind to the promoter region of cell-cycle regulator *cdk1* and Wnt pathway transcriptional and co-activator *earthbound1/Jerky* (*Ebd1*) in NSCs, suggesting that Pr-set7 is linked to gene activation in NSCs [42]. Both Cdk1 and Ebd1 are intrinsically required for NSC reactivation and the expression of these genes in NSCs is regulated by Pr-set7 [42]. Interestingly, Cdk1 and Ebd1 can also reciprocally regulate one another during NSC reactivation, where Cdk1 upregulates Ebd1 levels and Ebd1 downregulates Cdk1 levels, thus forming a negative feedback loop to maintain an equilibrium of Cdk1 and Ebd1 levels during NSC reactivation [42].

Ebd1 is a Wnt/Wingless signaling pathway transcriptional co-activator, and it stabilizes the Arm-TCF complex and facilitates the recruitment of the complex to chromatin [46]. Ebd1 localizes in the nucleus of quiescent and reactivating NSCs, in contrast to NSCs at third instar larvae where nuclear Ebd1 is absent [42, 46]. Thus, Ebd1 expression is developmentally regulated and the Wingless signaling pathway may be transiently activated at the early larval stage during NSC reactivation [42]. Pr-set7 promotes Ebd1 expression to activate Wnt/Wingless signaling, as the expression of Arm/β-catenin in NSCs in the early larval stage is also dependent on Pr-set7, highlighting the importance of Pr-Set7 in regulating the Wnt/Wingless pathway during neural development [42]. The role of other key components of the Wingless pathway during NSC reactivation is currently unknown and will be of great interest for future investigations.

## THE DYNAMICS OF CELLULAR PROTRUSION/BASAL FIBRE OF QUIESCENT NSCS

One distinct morphological feature of quiescent NSCs in *Drosophila* is their cellular extension(s) that is attached to the cell body. Quiescent NSCs in *Drosophila* extend a primary cellular protrusion/basal fibre toward the neuropil and occasionally extend a second but a much shorter protrusion at the opposite side of the cell body [15, 29]. This morphological feature shares great similarity with radial glia, a mammalian NSC type found in the developing brain [47]. Mammalian radial glial cells extend an apical process attached to the ventricular surface and a longer basal process extending toward the pial surface of the brain [47]. The basal process of radial glial cells has long been known to act as a scaffold, guiding the migration of newborn neurons to their correct position in the neocortex [67, 68]. Whether the fibres in radial glia are involved in quiescent NSC reactivation is currently unknown.

Are cellular protrusions of *Drosophila* quiescent NSCs similar to other microtubule-enriched signaling structures such as cilia or nanotubes? Microtubule-based nanotubes mediate signaling between *Drosophila* male germline stem cells and their niche [63]. However, the structure of the primary protrusion in qNSCs is distinct from that of nanotubes, as the latter lacks acetylated Tubulin and is much thinner and shorter. Furthermore, quiescent NSC primary protrusion is distinct from primary cilia, as the latter is assembled/attached from the basal body, which is derived from the mother centriole [64]. The primary protrusion of quiescent NSCs also differs from the cytonemes and tunneling nanotubes that were up to 700–1000 μm in length and mediated long-range signaling between cells [65, 66].

Cellular protrusions of quiescent NSCs are removed presumably via retraction prior to cell cycle re-entry [29]. If the fibre is retracted, is it a pre-requisite for quiescent NSCs to re-enter the cell cycle? Does the fibre in quiescent NSCs provide any cues for the apicobasal polarity of dividing NSCs? Interestingly, the centrosomes, although inactive (refer to the following section), are located at the apical side of the quiescent NSCs, away from the cellular protrusion [48]. The position of immature centrosomes in quiescent NSCs appears to mark the future apical side of the dividing NSCs. Further analysis is warranted to test whether the fibre provides the earliest cue for the apicobasal polarity of NSCs.

By using immobilization techniques to allow for long-term live imaging, Bostock et al. recently documented NSC reactivation in the larval ventral nerve cord at 22–24 h after larval hatching (ALH) [49]. In contrast to the previous notion, quiescent NSCs can retain their cellular protrusion throughout the first post-reactivation division, after which the cellular protrusion is inherited by the first newborn ganglion mother cell (GMC) and then by the GMC progeny in the following divisions [49]. This observation awaits further confirmation by analysing the dynamics of cellular protrusion of quiescent NSCs *in vivo* in intact larvae. The exact mechanism by which this inheritance occurs also remains to be elucidated. Can fiber retraction and inheritance occur in different populations of quiescent NSCs? Alternatively, the fibre might undergo an incomplete retraction before its inheritance by the daughter cell committed to differentiation. What is the significance of fibre inheritance by neuronal progeny? Does it play a role in synapse or circuit formation in the larval or adult central nervous system?

## NON-CENTROSOMAL MICROTUBULE GROWTH AND ORIENTATION IN QUIESCENT NSCS

Although the primary protrusion is believed to be a hallmark of quiescent NSCs, the exact structure and function in NSC reactivation are poorly studied. Recent work from our research group reported that these cellular extensions of quiescent NSCs are microtubule-enriched structures [50]. Are these microtubules organized by the centrosomes in quiescent NSCs? Surprisingly, in quiescent NSCs of newly hatched larvae, centrosomes are immature and lack microtubule-nucleation activity [49]. In centrosome-deficit quiescent NSCs, microtubule growth is still robust, indicating that microtubule growth in the primary protrusion of quiescent NSCs is mostly acentrosomal. The γ-tubulin ring complex (γ-TuRC) including γ-tubulin is often required for acentrosomal microtubule nucleation in tracheal, wing and salivary gland epithelia as well as neurons [51–54]. Super-resolution imaging in *Drosophila* quiescent NSCs indicates that γ-tubulin is localized to the centrioles, but not pericentriolar material [49], suggesting that microtubule assembly in quiescent NSCs likely occurs independently of γ-TuRC.

Mini spindles (Msps)/MAP215 has been identified as the first key regulator of acentrosomal microtubule growth in the primary protrusion of quiescent NSCs [49]. Msps/MAP215 directly binds to the tubulin dimer via its tumor-overexpressed gene (TOG) domains to promote microtubule polymerization [50]. In quiescent NSCs upon *msps* depletion, very little growing microtubules can be detected in the cellular protrusion, indicating a critical role of Msps in promoting the microtubule polymerization in quiescent NSCs [49]. Interestingly, Msps-dependent microtubule growth also provides structural support for the formation of the primary protrusion, as *msps* depletion leads to the thinning of the fibre of quiescent NSCs [49].

Microtubules are inherently polarized, possessing a faster-growing plus end and a slower-growing minus end. Our recent work has discovered that acentrosomal microtubules within the cellular protrusion are oriented predominantly plus-end-out, similar to that seen in axons of vertebrate and invertebrate neurons [49]. This plus-end-out orientation is altered in quiescent NSCs upon *msps* depletion [49], consistent with the role of Msps in regulating minus-end microtubule orientation in *Drosophila* dendrites in sensory neurons [55]. It remains to be tested whether microtubule misorientation in *msps* mutants is caused by the rotation of shorter microtubules that presumably resulted from decreased microtubule polymerization and, in turn, the switch of microtubule orientation. Interestingly, microtubules in the basal process of mouse apical radila glia appear largely acentrosomal with approximately 85% of them oriented plus-end-out [69], analogous to that of the primary protrusion of quiescent NSCs in *Drosophila*. What is the significance of the plus-end-out microtubule orientation in quiescent NSCs? It is conceivable that plus-end-out microtubule orientation might be important for the transport of cargos such as proteins, organelles and vesicles, in the fibre of quiescent NSCs. As the microtubule polarity has just beginning to be elucidated in both flies and mammalian NSCs, future studies on potential acentrosomal microtubule-organizing centre in quiescent NSCs as well as conserved mechanisms that regulate microtubule polarity in both flies and mammalian NSCs are warranted.

## NSC-NEUROPIL CONTACTS AND CELL ADHESION MOLECULES IN QUIESCENT NSCS

*Drosophila* quiescent NSCs extend their primary cellular protrusion towards the neuropil [15, 29]. Recent work using Targeted GFP Reconstitution Across Synaptic Partners (t-GRASP), has shown that quiescent NSCs directly contact the neuropil [49]. The t-GRASP method has previously been shown to specifically detect cell–cell interactions including those in synapse formation [56]. Using two split-GFP fragments, one split-GFP fragment is targeted specifically to the NSCs, while the other split-GFP fragment is targeted specifically to the neuropil, the full-length GFP could be successfully reconstituted at the extracellular space between the two cell types, marking the membrane contact sites between quiescent NSCs and the neuropil [49]. The following open questions remain to be answered—Can neuropil function as a new niche to control NSC quiescence and reactivation? Which signaling pathways are mediated by the NSC-neuropil contacts in quiescent NSCs?

What are proteins that facilitate NSC-neuropil communication? The cell adhesion molecule E-cad that is often localized to cell–cell contacts forms endfeet-like structures at NSC-neuropil contact sites and is intrinsically required for NSC reactivation [49]. *Drosophila* E-cad, together with β-catenin and α-catenin, forms adherens junctions in epithelial cells and regulates cell adhesion and the apicobasal polarity of epithelial cells [57]. Furthermore, E-cad has also been reported to localize to MB NSC-cortex glia contact in the adult *Drosophila* brain [58, 59]. How exactly is E-Cad transported to these NSC-neuropil contact sites? The localization of E-cad at NSC-neuropil contact sites is possibly mediated by Msps-dependent microtubule growth with the help of the microtubule plus-end-directed motor protein, Kinesin-2 [49]. Both Msps and Kinesin-2 play a critical role in promoting quiescent NSC reactivation [49], suggesting that plus-end-oriented cargo transport might be required for NSC reactivation. Several studies have shown microtubule-dependent transport of E-cad in various cell types, such as in the elongated epidermal cells within the *Drosophila* embryo [60]. In HeLa cells, overexpressed E-Cad was found to transit from the Golgi to the Rab11 endosomes [61]. In *Drosophila* epithelial cells, E-cad is delivered from recycling endosomes to the plasma membrane [62]. It remains to be determined whether the localization of E-cad at the NSC-neuropil contact sites is mediated by membrane trafficking in quiescent NSCs. It will also be important to identify additional molecules that are localized and function at the NSC-neuropil contact sites.

## CONCLUSIONS AND FUTURE PERSPECTIVES

*Drosophila* is indeed an indispensable model system for understanding molecular mechanisms regulating NSC quiescence and reactivation *in vivo*. As discussed in this review, recent findings have opened new avenues into the understanding of epigenetic regulation as well as the structure and function of quiescent NSCs cellular protrusuions in reactivation of quiescent NSCs. The primary protrusion of quiescent NSCs arises as novel microtubule enriched, signaling structure that possibly mediates NSC-neuropil communication, contributing to timely reactivation of *Drosophila* quiescent NSCs. Future studies on novel mechanisms underlying epigenetic regulation and microtubule-dependent transport during NSC reactivation in *Drosophila* and mammalian systems will provide insights into how these regulators modulate stem cell behaviour in a more complex system, with important implications in understanding neurological disorders and potential therapeutic targets.

## SUPPLEMENTARY MATERIAL

Supplementary material are available at *Oxford Open Neuroscience* online.

## Conflict of interest

None declared.

## Supplementary Material

suppl_data_kvac001
