# Supplementary material for: A fly’s eye view of quiescent neural stem cells
Source: Oxf Open Neurosci. 2022 May 4;1:kvac001. doi: 10.1093/oons/kvac001 (PMC10913722; doi:10.1093/oons/kvac001)
Supplement: suppl_data_kvac001 [file suppl_data_kvac001.zip › kvac001_REVIEWER COMMENTS oxfnsc-2021-004.docx]

REVIEWER COMMENTS

OXFNSC-2021-004 A Fly's Eye View of Quiescent Neural Stem Cells

Original submission

Reviewer 1

Review of “A Fly's Eye View of Quiescent Neural Stem Cells” by Carlos Ibanez

This is a timely, interesting, and well-written short piece summarizing recent discoveries of epigenetic and cytoskeleton regulatory mechanisms which impact on the balance of quiescence versus activation in neural stem cells of Drosophila. The review does a good job at posing numerous questions for future study along the way. I recommend the piece for publication and present below a few suggestions for the authors to consider. 

1. This review is about insect (Drosophila) NSCs, yet the Abstract opens with statements about mammalian NSCs, giving the impression that these will be the main topic covered. Not until the last sentence of the Abstract does the reader find out that the piece is actually about Drosophila. It feels a bit strange to set the stage using mammalian examples if the main discussion will be about insect NSCs. May be this could be rephrased. 
2. The terms “activation” and “reactivation” seem to be used interchangeably. May be this can be clarified. Also, it is unclear here whether “reactivation” just means re-entering the cell cycle or includes in addition further differentiation into and generation of mature neurons. Presumably, quiescent NSCs could re-enter the cell cycle, divide a few times, and this progeny become quiescent again. Such a “reactivation” would not result in any new mature neurons. Perhaps this could be specified a bit more. 
3. BBB (line 40) should be spelled out here. 
4. It is stated (lines 45 and 46) that “The function of IIS in growth is evolutionarily conserved in Drosophila”. It is unclear what is being meant by this statement. A trait or function can be evolutionarily conserved along a lineage, or between two or more organisms. To say that something is evolutionarily conserved in Drosophila would seem to suggest that the authors refer to the species that comprise the Drosophila genus, when I assume they are actually comparing the IIS pathway in flies with that of mammals. If the case is indeed the latter, they could perhaps consider indicating this more clearly. 
5. “Cellular protrusions” (eg line 115) are mentioned. Are these equivalent to cytonemes or distinct?
6. ALH (line 130) should be spelled out here. 
7. The final, two sentence-long section titled “Conclusions and future perspectives” is far too general and vague, merely stating the obvious (eg Drosophila is an important model, further studies will provide more insights, etc). It does not provide the kind of ending that such an otherwise interesting and well-written piece actually deserves. The authors may wish to consider providing a more substantial summary of the main takes regarding epigenetic and microtubule mechanisms for NSC reactivation, as this is not provided in the Abstract. It may also be useful here to spell out some of the most important problems to be tackled in future research in this area (from the many that were already mentioned, but which remain scattered around, in the text).

Reviewer 2

Neural stem cells are important for the development and regeneration of the nervous system. In this current review, the author summarizes the recent advances in epigenetic regulations and microtubule cytoskeleton. overall, this is well-written review which highlights some aspects of the field of neural stem cell. Only one suggestion is that the author may discuss and elaborate the structural similarity and difference between quiescent fly and mammalian NSCs. Do mammalian quiescent NSCs have similar protrusions? also their potential involvement in reactivation?

Decision letter

13-Jan-2022 

Dear Hongyan, 

Hope all is well. Your manuscript ID OXFNSC-2021-004 entitled "A Fly's Eye View of Quiescent Neural Stem Cells" which you submitted to Oxford Open Neuroscience, has been reviewed by myself and one anonymous reviewer.  The comments are included below. The reviewer(s) have recommended publication, but also suggest some minor revisions to your manuscript. These are suggestions; I encourage you to consider them but will leave it to your own discretion to do so.  

No rebuttal is needed. You may want to quickly summarize what changes (if any) you made in a cover letter. Once you are happy with a final version of your manuscript, simply submit it using this link: 
https://mc.manuscriptcentral.com/oxfnsc?URL_MASK=d0a92051862944eab05d5e542d3e1b81 

Best regards, 

Carlos

Author response

Thank you very much for the rapid review process and constructive comments from both reviewers to improve our manuscript. Please find enclosed our revised manuscript entitled “A Fly's Eye View of Quiescent Neural Stem Cells”, which we submit for publication as a Review article in Oxford Open Neuroscience. 

The revised manuscript has addressed all concerns raised by both reviewers. We have revised our manuscript from the abstract, main text, to the concluding paragraph. We believe the revised manuscript is much improved and appropriate for publication in Oxford Open Neuroscience. 

We have also uploaded the revised manuscript with all changes tracked, for your convenience.

Revised submission

Immediate accept on revision no further AE comments.

Decision letter

19-Jan-2022 

Dear Hongyan, 

It is a pleasure to accept your revised manuscript entitled "A Fly's Eye View of Quiescent Neural Stem Cells" in its current form for publication in Oxford Open Neuroscience.  

Best regards,  

Carlos
